# Investigation of chitin grafting: thermal, antioxidant and antitumor properties

**DOI:** 10.1186/s11671-025-04185-y

**Published:** 2025-01-15

**Authors:** Nevin Çankaya, Mehmet Mürşit Temüz, Burak Can

**Affiliations:** 1 Vocational School of Health Services-Oral and Dental Health Department, Usak University, 64200 Uşak, Turkey; 2 Faculty of Science, Department of Chemistry, Firat University, 23200 Elazığ, Turkey; 3 Institute of Science, Department of Chemistry, Firat University, 23200 Elazığ, Turkey

**Keywords:** Chitin, Graft copolymer, Thermal stability, Antioxidant, Antitumor

## Abstract

In this study, firstly chitin was reacted with chloracetyl chloride to synthesize the macroinitiator chitinchloroacetate (Ch.ClAc). Then, graft copolymers of methacrylamide (MAM), diacetone acrylamide (DAAM), N-(4-nitrophenyl)acrylamide (NPA), and 2-hydroxyethyl methacrylate (HEMA) monomers were synthesized by atom transfer radical polymerization (ATRP). All of the polymers were characterized by FTIR spectra and elemental analysis. According to the elemental analysis results, the mole percent (y) of the macro initiator was found to be 17.39%. The thermal stability of all the polymers (chitin, Ch.ClAc and its graft copolymers) was determined by thermogravimetric analysis (TGA) method and the highest thermal stability was observed in the ungrafted raw chitin. DPPH• scavenging activity and antitumor activity of all polymers were then investigated. Ch.ClAc was found to be the polymer that inhibited the proliferation of tumor cells more than chitin and graft copolymers. It was observed that the antitumor (L1210 cell lines) effect increased with increasing time and concentration in all polymers.

## Introduction

Chitin is one of the abundant biopolymers in nature and can be found in the exoskeleton of crustaceans and in the cell walls of fungi, insects and yeast [1]. Chitin and its derivatives are used in many different fields such as wastewater treatment, textiles and paper, cosmetics, food and health supplements, agriculture, agrochemical use and biotechnology [2, 3]. It also has various biological activities due to its biodegradability and biocompatibility. It can be biodegraded in the human body, has immunological, antibacterial, and wound healing properties [1]. In fact, many types of materials containing chitin have been patented for use in wound dressing applications chitin and chitosan (deacetylated form of chitin) have been proven to have some biological activities such as antitumor, antimicrobial and antifungal [4, 5]. In the current study, we investigated the antioxidant and antitumor properties of chitin and its derivatives.

ATRP (Atom transfer radical polymerization), a multicomponent polymerization method, is a catalyst system consisting of a monomer, an initiator consisting of an alkyl halide with a transferable halogen and a transition metal with a suitable ligand. ATRP is a controlled-living polymerization method [6, 7]. Since polymer chains grow in a controlled manner in ATRP, it is a very useful method in the synthesis of block and graft copolymers [8]. Another advantage compared to traditional radical initiators is that almost no homopolymerization occurs in graft copolymerization [9]. For this reason, the grafting effect of ATRP on solid surfaces is quite good [10].

In this study, our first objective was to synthesize and characterize a chitin macroinitiator as ATRP initiator for the first time in the literature. Then, we synthesized synthesised graft copolymerization by interacting different amide monomers with this newly synthesized chitin initiator. We performed structural characterizations of the synthesised polymers and investigated their thermal stability. We investigated the antioxidant and antitumor activities of chitin, whose biological activity is known in the literature. Similar to the literature, while chitin itself did not show antioxidant properties, the Ch.ClAc initiator synthesized for the first time showed good DPPH• scavenging activity. In our antitumor study on L1210 murine leukemia cells, the macroinitiator again showed the best property.

## Experimental

### Instruments and materials

FTIR spectra of all polymers were measured with a Mattson 1000 FTIR spectrometer. The elemental analysis was performed using Leco CHNS-932. Using a Shimadzu TGA-50 thermobalance, thermal analysis was carried out at a heating rate of 10 °C/min and a N_2_ flow rate of 10 mL/min.

For this study, crude chitin, potassium-t-butylate, chloroacetyl chloride were purchased from Aldrich. Then, copper(I) chloride, 2,2′- bipyridine (Aldrich) as catalysts; methacrylamide (MAM), diacetone acrylamide (DAAM), N-(4-nitrophenyl)acrylamide (NPA), and 2-hydroxyethyl methacrylate (HEMA) (Aldrich) were used as monomer. NPA monomer was resynthesized by our team for this study [10]. DMF, acetonitrile, ethanol, acetone, and ether were used as solvent.

### Synthesis of chitinchloroacetate

**Fig. 1:**
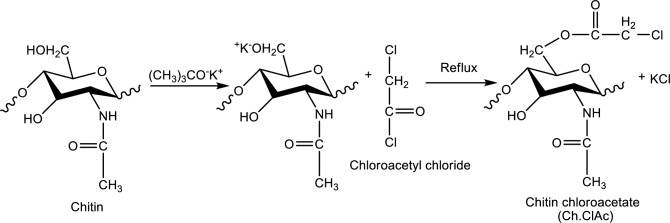
Synthesis of chitinchloroacetate macroinitiator by modifying chitin

4.0 g of crude chitin was stirred in 50 mL acetonitrile overnight. 0.07 mol potassium-*t*-butylate dissolved in acetonitrile was added to the swollen chitin and stirred for 4 h. 0.2 mol chloracetyl chloride dissolved in acetonitrile was added dropwise. The reaction was refluxed for 36 h. The mixture was filtered and washed in water, ethanol, acetone and ether to remove any impurities and dried in a vacuum oven. Methods that were modified from the literature were used to synthesis Ch.ClAc and its graft copolymers [12–15]. It was sieved through a 10 micron sieve and partially brought to nano size. The formation reaction of synthesized Ch.ClAc is shown in Fig. 1.

### Synthesis of chitin graft copolymers

**Fig. 2:**
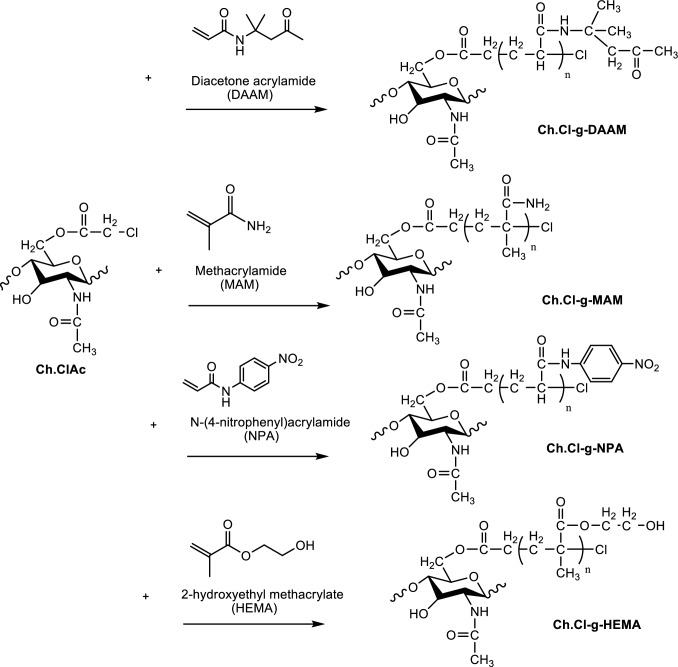
Synthesis of graft copolymers

The calculated amount of 2,2′-bipyridine and CuCl was passed through argon gas in the polymer tube. Ch.ClAc macroinitiator and 10 mL DMF solvent with monomer were added and argon gas was passed for 15 min. The polymer tube was sealed and grafting was carried out at 130 °C for 24 h with continuous stirring. The amount of material in the graft polymerization was calculated based on the Cl content in moles in Ch.ClAc. CuCl, 2,2′-bipyridine, and amide monomers (MAM, DAAM, NPA, and HEMA) were used. Mole ratios in polymerization were calculated as 1/1/3/60 respectively [12–15]. The mixture was cooled, filtered, and the precipitate was washed with DMF, acetonitrile, chloroform, water–ethanol-HCl mixture, water, acetone and ether to remove oligomers and homopolymers formed in the reaction. All polymers were sieved through a 10 micron sieve and partially brought to nano size. The reaction of the grafting of Ch.ClAc with MAM, DAAM, NPA, and HEMA monomers is shown in Fig. 2.

### Free radical (DPPH) scavenging activity of the chitin and its polymers

DPPH (2,2-diphenyl-1-picrylhydrazyl) radical scavenging method was used to determine antioxidant activity. 4 mL of 1 mM DPPH solution was added to each test tube. After adding 5 mg of polymer, the mixture was incubated at room temperature and in the dark for 30 min. Absorbance was measured at 517 nm in relation to the ethanol blank following incubation. 4 mL DPPH solution was used as a control, and ethyl alcohol was used as a blank. The amount of DPPH radicals eliminated by the samples from the environment was calculated according to the literature [16–19].

### Antitumor activity study of chitin and its polymers

The American Type Culture Collection (ATTC) provided the L1210 murine leukemia cell line. 75 cm^2^ flasks containing 5% CO_2_ and 37 °C were used to cultivate L1210 cell lines. Cells were checked continuously with an inverted microscope at three-day intervals. After the fifteenth day, the medium was taken out of the flasks and replaced with three milliliters of trypsin before being put back into the incubator. Trituration was performed to ensure that the cells were homogeneously distributed in the solution, and the cells were counted with a hemocytometer. Class II laminar flow was used to maintain sterility during cell culture, feeding, and experimentation [20, 21]. L1210 murine leukemia cells were centrifuged (2000 rpm) by adding trypsin, trituration was performed, and cells were counted with a hemocytometer. For the purpose of analyzing the test compounds' antitumor properties, six duplicates of 1 × 10^6^ cells/mL L1210 cells per eppendorf tube were seeded for the trypan blue test. The test chemicals' solvent of choice was DMSO. After being planted into flasks, live L1210 cells were cultured for a full day. Following pre-incubation, test compounds produced at doses of 7.5, 15, 30, and 60 μM were added to the cell culture medium, which was then replaced with new medium. The trypan blue exclusion method was used to assess the vitality of cells cultured at 37 °C and 5% CO_2_ for 24 and 48 h (1:1 (v/v)) [17, 22, 23]. The DMSO concentration in the cell growth media was found to be less than 1%, even in the vehicle-treated tubes that were used as controls.

### Statistical analysis

The statistical package SPSS 15.0 was used to analyze the antitumor activity experiment data, and the mean value ± standard deviation is shown as the result. Analysis of One-way Anova and Tukey test were used to investigate the significance of differences between group means. P < 0.05 values in the statistical results were regarded as significant.

## Result and discussion

### Grafting of chitin and its characterization

Chloroacetyl group with esteric structure is known to be an effective initiator for ATRP. In this study Ch.ClAc, which can be used as an ATRP macroinitiator by grafting chitin, was synthesized for the first time in the literature. Although an excess of potassium-*t*-butylate is used in the reaction of chitin with potassium-*t*-butylate, only the alkoxide of chitin at the primary OH group is produced. By reacting chloroacetylchloride with potassium chitin alkoxide, Ch.ClAc is synthesized [12–15].

### FTIR characterization of the polymers

**Fig. 3:**
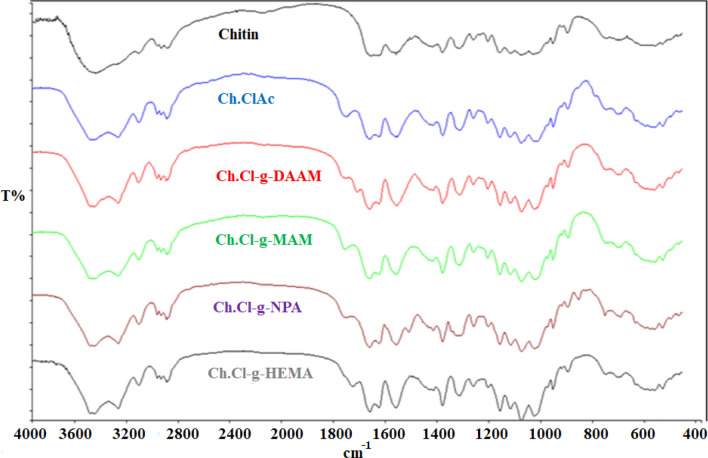
FTIR spectra of chitin, chitinchloroacetate and its graft copolymers

Looking at the FTIR spectra of all polymers containing chitin, O–H stretching peaks in the region of approximately 3350 cm^−1^ can be clearly observed [2, 24]. The presence of C=O ester stretching peaks observed in Ch.ClAc and its graft copolymers and the fact that this band was not observed in chitin is evidence that the chloroacetyl group is chemically bound to chitin [2, 12–15]. Another evidence showing that the amide groups present in the monomer structure are grafted onto the Ch.ClAc macroinitiator is the C=O amide stretching peaks observed at 1660 cm^−1^. In addition, the results calculated by gravimetric analysis were another indicator that graft copolymers were formed. The FTIR spectra of chitin, Ch.ClAc and graft copolymers are shown in Fig. 3, and their interpretations are presented in Table 1.

**Table 1:** FTIR spectra results of chitin, chitinchloroacetate and its graft copolymers

Polymer	Wavenumbers ~ (cm^−1^)	Vibration types
Chitin	3350 1670	O–H stretching C=O amide stretching
Ch.ClAc	3350 1745 794	O–H stretching C=O ester stretching C–Cl stretching
Ch.Cl-g-DAAM	3350 1740 1660	O–H stretching C=O ester stretching C=O amide stretching
Ch.Cl-g-MAM	3350 1722 1665	O–H stretching C=O ester stretching C=O amide stretching
Ch.Cl-g-NPA	3350 1722 1665 1320	O–H stretching C=O ester stretching C=O amide stretching NO_2_ stretch
Ch.Cl-g-HEMA	3350 1745 750 1660	O–H stretching C=O ester stretching C–Cl stretching C=O amide stretching

### Elemental analyses of the polymers

The (x) and (n) values and elemental analysis results calculated according to the elemental analysis C% result of chitin, chitinchloroacetate and graft copolymers are given in Table 2. The substitution percentage of glucose units of chitin was adapted from the literature and calculated as percent by weight (Y), 22.32% [2, 12–15, 25]. Then, the following formula was used to calculate mole percent substitution (y), 17.39%. In this formula (Eq. 1), 203 refers to the unit weight of glucosamine repeating in chitin, and 279.50 refers to the unit weight of Ch.ClAc macroinitiator when 100% substitution occurs.

**Table 2:** Elemental analyses results and evaluation of chitin, chitinchloroacetate and its graft copolymers

Polymer	Elemental analyses			Weight fraction of monomers (x)	Number of monomer units per macroinitiator (n)
	C%	H%	N%		
Chitin	43.93	6.29	6.63	–	–
Ch.ClAc	36.25	5.20	5.40	–	–
Ch.Cl-g-DAAM	47.65	6.65	6.87	0.41	0.36
Ch.Cl-g-MAM	44.85	6.47	7.28	0.43	0.77
Ch.Cl-g-NPA	47.80	5.75	9.22	0.54	0.56
Ch.Cl-g-HEMA	45.69	6.70	6.17	0.49	0.66

1 $$\text{y}= \frac{\text{Y}/279.5}{\frac{\text{Y}}{279.5}+\frac{100-\text{Y}}{203}/}.100$$

The following formula (Eq. 2) was used to calculate the weight fraction (x) of amide monomers in each of the grafted chitins. In this formula, E is the experimental carbon percentage of graft copolymers; F is the theoretical carbon percentage of monomers; A refers to the experimental carbon percentage of Ch.ClAc.

2 $$\text{E}=\left(1-\text{x}\right).\text{A}+\text{F}.\text{x}$$

The ratio (n) of amide monomer unit to Ch.ClAc macroinitiator unit in graft copolymers was calculated using the formula below (Eq. 3). Mm refers to the molecular mass of the monomer, and Mavg refers to the degree of substitution of the average molar mass of the chitin unit [2, 12–15, 25].

3 $$\text{n}= \frac{\text{x}/\text{Mm}}{(1-\text{x})/\text{Mavg}}/\text{ y}$$

### Thermogravimetric characterization of the polymers

**Fig. 4:**
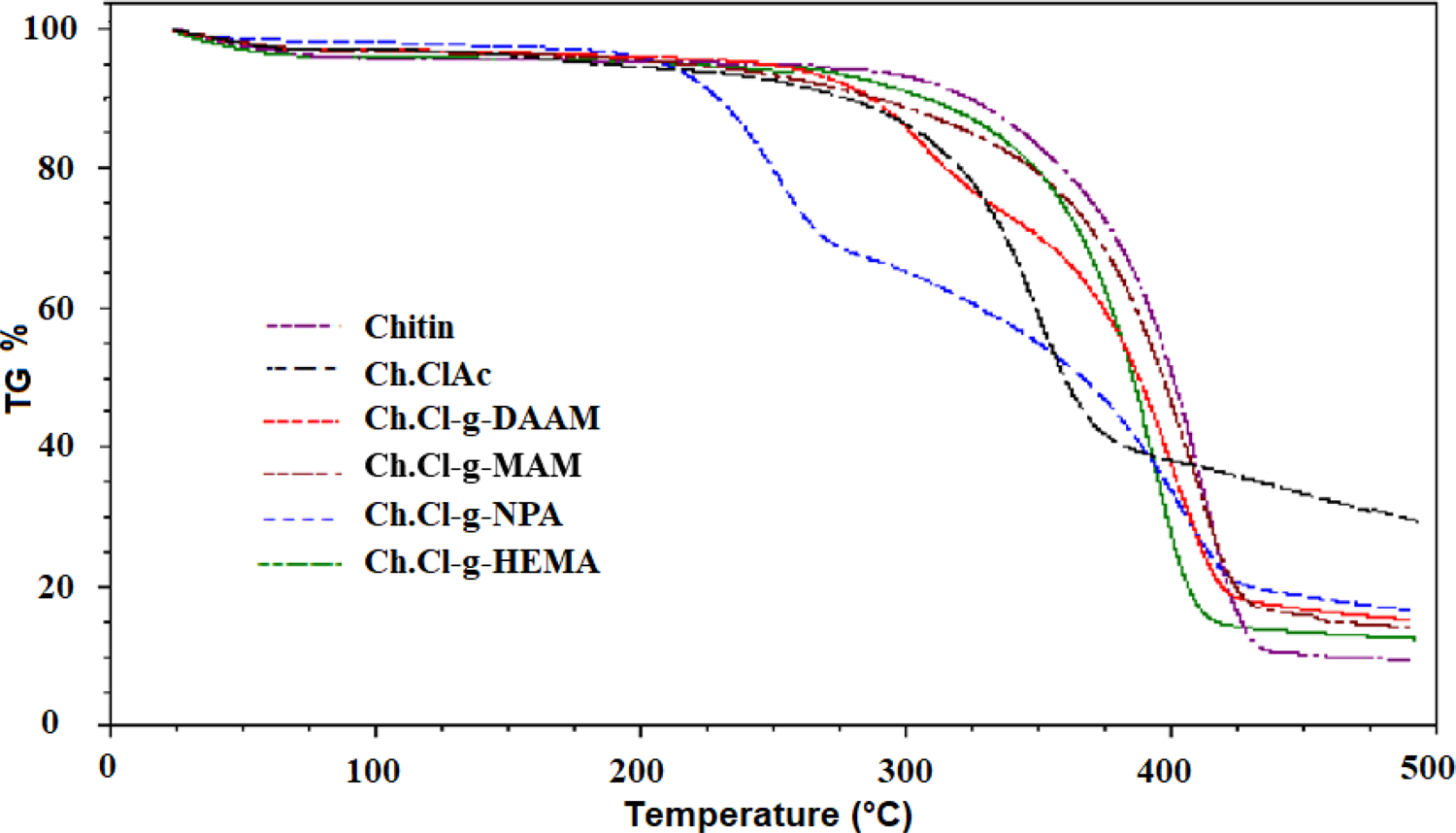
TGA curves of chitin, chitinchloroacetate and its graft copolymers, comparatively

The thermal stability of unprocessed and raw natural polymers is higher than that of semi-synthetic natural polymers. Similar results are known in the literature for both chitin and other natural polymers [2, 3, 12–15]. In previous studies, our team had observed that the max decomposition temperature of chitin was higher than that of grafted chitin polymers. In this study, the thermal stability of crude chitin was higher than other grafted polymers. The residue left by chitin after heat combustion was found to be less than that of grafted chitin polymers, and our team has already obtained similar results. While the degradation of chitin was observed in a single step and over a narrow temperature range, the degradation of graft copolymers occurs in two steps and the range is widened. This may be related to some cross-linking reactions during degradation. The thermal decomposition temperature range of chitin is in accordance with the range in the literature [2, 3]. Tanodekaew et al. treated chitin with acrylic acid and reported that the bonds between acrylic acid and chitin probably reduce the thermal stability of chitin [3]. In this study, the chloroacetate groups in Ch.ClAc used as macroinitiator decreased the thermal stability and thus may decreased the thermal stability of the graft copolymers. TGA (Thermogravimetric analysis) curves of chitin, Ch.ClAc and graft copolymers are shown comparatively in Fig. 4, and their some thermal analyses results are presented in Table 3.

**Table 3:** Thermal analyses results of chitin, chitinchloroacetate and its graft copolymers

Polymer	Initial decomp. temp. (^o^C)	Temp. of 50% weight loss (°C)	Residue% (500 oC)
Chitin	305	405	8
Ch.ClAc	270	370	30
Ch.Cl-g-DAAM	275	395	15
Ch.Cl-g-MAM	295	400	14
Ch.Cl-g-NPA	220	375	18
Ch.Cl-g-HEMA	280	385	12

### Free radical (DPPH^•^) scavenging results of polymers

One of the properties of antioxidant substances is that they scavenge radicals formed in the environment [26–28]. Many antioxidants are also antiradical. These antioxidants neutralize radicals by pairing their unpaired electrons. According to the DPPH^•^ scavenging activity results, it is observed that the free radical scavenging activities of the polymers are low compared to pyrocatechol, which is considered a standard antioxidant [19, 22]. Depending on the amount of oxidant, it can be seen in Table 4 that Ch.ClAc has a moderate antioxidant effect and graft copolymers have a low antioxidant effect. It has been observed that chitin has no antioxidant effect. The DPPH^•^ scavenging activities of the polymers are listed from largest to smallest as follows: Ch.ClAc > Ch.Cl-g-DAAM > Ch.Cl-g-MAM > Ch.Cl-g-NPA > Ch.Cl-g-HEMA > Chitin (Table 4).

**Table 4:** DPPH^•^ scavenging activities of chitin, chitinchloroacetate and its graft copolymers

Polymers (5 mg)	DPPH^•^ scavenging activity (%)
(–) Control	0.00
Chitin	0.00
Ch.ClAc	56.95
Ch.Cl-g-DAAM	15.19
Ch.Cl-g-MAM	9.03
Ch.Cl-g-NPA	7.97
Ch.Cl-g-HEMA	5.24
(+) Control pyrocatechol	85.24

Kaur et al., by optimizing the production of chitin from the fungus *Aspergillus niger*, demonstrated that the produced chitin had remarkable DPPH antioxidant potential [29]. Limam et al. examined the antimicrobial and antifungal properties of chitin and chitosan [30]. They found that Parapenaeus longirostris chitin and Parapenaeus longirostris chitosan had good levels of DPPH scavenging activity, and Squilla mantis chitin had nearly good levels of DPPH [30]. Kong et al. synthesized the water-soluble derivatives carboxymethyl (CM)-chitosan and CM-chitin and suggested that they are potent antioxidants and matrix metalloproteinase inhibitors by mitigating radical-induced oxidative damage [31]. Yanat et al. found that the chitin nanocrystals they produced through acid hydrolysis showed more DPPH^•^ scavenging activity compared to raw chitin powder [32].

### Antitumor activity assays of polymers

Live cell results of L1210 cells treated with polymers are given in Table 5. When the antitumor results of chitin and the control group are compared, it is observed that chitin has an antitumor effect depending on time and concentration (9.50% for 48 h and 5 mg). The antitumor activities of different chitin derivatives have been investigated in the literature [33, 34]. Song et al. proved the antitumor activities of CM-chitin and N-succinyl-chitosan against L1210 leukemia [35]. Nishimura et al. discovered that CM-chitin and partially deacetylated chitin were useful in suppressing different tumor cells [36]. Ohya et al. investigated the in vivo and in vitro antitumor activities of 6-O-CM-chitin [37]. According to Murata et al., the degree of sulfation in 6-O-sulfated chitin directly correlated with its ability to considerably prevent lung tumor colonization [34]. Moreover, in the spontaneous lung metastasis paradigm, 6-O sulfated CM-chitin significantly reduced the number of lung tumor colonies. Significant inhibition of B16Bl6 cell arrest in the lungs following co-injection with radiolabeled tumor cells was also observed with 6-O sulfated CM-chitin [1]. Chitin and chitosan derivatives of some anticancer agents and their conjugates show important distribution in cancer tissue. Release of the drug was achieved from these conjugates, gradually. Thus, its side effects were reduced compared to the original form and it was reported to have an anticancer effect [1].

**Table 5:** Antitumor activity results of chitin, chitinchloroacetate and its graft copolymers

Polymers	24 h and 2.5 mg (cell viability %)	24 h and 5 mg (cell viability %)	48 h and 2.5 mg (cell viability %)	48 h and 5 mg (cell viability %)
Control	88.00 ± 0.70	87.00 ± 0.41	83.75 ± 0.47	82.25 ± 0.63
Chitin	25.75 ± 1.37*	16.00 ± 0.40*	14.25 ± 1.10*	9.50 ± 0.50*
Ch.ClAc	8.00 ± 0.91*	0.00 ± 0.00*	0.00 ± 0.00*	0.00 ± 0.00*
Ch.Cl-g-DAAM	26.50 ± 1.55*	18.75 ± 0.47*	14.50 ± 0.64*	2.00 ± 0.40*
Ch.Cl-g-MAM	30.25 ± 1.25*	20.25 ± 0.47*	17.50 ± 1.32*	8.50 ± 1.04*
Ch.Cl-g-NPA	15.75 ± 0.47*	7.25 ± 0.63*	0.00 ± 0.00*	0.00 ± 0.00*
Ch.Cl-g-HEMA	35.50 ± 1.32*	26.50 ± 0.95*	15.00 ± 1.78*	7.00 ± 0.70*

*P < 0.001

In our study, Ch.ClAc, a chitin derivative, showed a significant antitumor effect depending on time and concentration. Graft copolymers were observed as Ch.Cl-g-DAAM: 2.0, Ch.Cl-g-MAM: 8.5, Ch.Cl-g-NPA: 0.0 and Ch.Cl-g-HEMA: 7.0 at 48 h and 5 mg. In addition, when these graft copolymers are compared with the control, it is observed that they give good antitumor results depending on time and concentration. As a result, when looking at all polymers, the antitumor effect increases in direct proportion to time and concentration.

## Conclusion

In this study, the macroinitiator chitinochloroacetate (Ch.ClAc) was synthesized from the reaction of chitin with chloracetyl chloride. Graft copolymerization were performed with methacrylamide (MAM), diacetone acrylamide (DAAM), N-(4-nitrophenyl)acrylamide (NPA), and 2-hydroxyethyl methacrylate (HEMA) monomers by ATRP. Each polymer was characterized by FTIR spectra and elemental analysis. From the elemental analysis results, the mole percent substitution (y) of the macroinitiator was found to be 17.39%. The thermal stability of the polymers was determined by TGA method and compared with each other. Some basic thermal values such as initial decomposition temperature and 50% weight loss at temperature decreased with grafting, and the highest thermal stability was observed in chitin. Then, DPPH• scavenging activity and antitumor activity of chitin, Ch.ClAc and its graft copolymers were investigated. The macroinitiator Ch.ClAc was detected to have the highest DPPH• scavenging activity. The macroinitiator Ch.ClAc was found to have the highest antioxidant and antitumor (L1210 cell line) properties. We think that this article, which includes the new synthesis and biological studies of chitin biopolymer-containing macroinitiator and its graft copolymers, will attract the attention of all researchers who want to study biopolymers and semi-synthetic polymers.

## Data Availability

Data is provided within the manuscript. The data underlying this article will be shared on reasonable request to the corresponding author.

## References

[1] Karagozlu MZ, Kim S-K. Anticancer effects of chitin and chitosan derivatives. Adv Food Nutr Res. 2014;72:215–25. 10.1016/B978-0-12-800269-8.00012-9. 25081085 10.1016/B978-0-12-800269-8.00012-9

[2] Çankaya N. Grafting studies of chitin. Sigma J Eng Nat Sci. 2019;37(1):111–7.

[3] Tanodekaew S, Prasitsilp M, Swasdison S, Thavornyutikarn B, Pothsree T, Pateepasen R. Preparation of acrylic grafted chitin for wound dressing application. Biomat. 2004;25:1453–60. 10.1016/j.biomaterials.2003.08.020 14643620

[4] Pae H-O, Seo W-G, Kim N-Y, Oh G-S, Kim G-E, Kim Y-H, Kwak H-J, Yun Y-G, Jun C-D, Chung H-T. Induction of granulocytic differentiation in acute promyelocytic leukemia cells (HL-60) by water-soluble chitosan oligomer. Leuk Res. 2001;25(4):339–46. 11248331 10.1016/s0145-2126(00)00138-7

[5] Hirano S. Chitin biotechnology applications. Biotechnol Annu Rev. 1996;2:237–58. 9704098 10.1016/s1387-2656(08)70012-7

[6] Wang J-S, Matyjaszewski K. Controlled/"Living" radical polymerization Halogen atom transfer radical polymerization promoted by a Cu(I)/Cu(II) redox process. Macromol. 1995;28(23):7901–10.

[7] Bhattacharjee M, Pramanik NB, Singha NK, Haloi DJ. Recent advances in RDRP-modified chitosan: a review of its synthesis, properties and applications. Polym Chem. 2020;11(42):6718–38.

[8] Liu S, Sen A. Syntheses of syndiotactic-polystyrene-graft-poly(methyl methacrylate), syndiotactic-polystyrene-graft-poly(methyl acrylate), and syndiotactic-polystyrene-graft-atactic-polystyrene with defined structures by atom transfer radical polymerization. Macromol. 2000;33(14):5106–10.

[9] Timothy VW, Patten TE. Preparation of structurally well-defined polymer−nanoparticle hybrids with controlled/living radical polymerizations. J Am Chem Soc. 1999;121(32):7409–10.

[10] Bicak N, Ozlem M. Graft copolymerization of butyl acrylate and 2-ethyl hexyl acrylate from labile chlorines of poly(vinyl chloride) by atom transfer radical polymerization. J Polym Sci A Polym Chem. 2023;41(21):3457–62.

[11] Tanış E, Çankaya N, Yalçın S. Synthesis, characterization, computation of global reactivity descriptors and antiproliferative activity of N-(4-nitrophenyl)acrylamide. Russ J Phys Chem B. 2019;13(1):49–61.

[12] Çankaya N. Modification of starch: structural and antimicrobial properties. Int J Chem Chem Eng Syst. 2018;3:31–5.

[13] Temüz MM, Çataldaş E. Investigation of chitosan grafting and uptake properties of some metal ions by atomic absorption spectrophotometry. J Macromol Sci B Phys. 2024. 10.1080/00222348.2024.2310438.

[14] Çankaya N, Temüz MM, Yakuphanoglu F. Grafting of some monomers onto cellulose by atom transfer radical polymerization Electrical conductivity and thermal properties of resulting copolymers. Cellul ChemTechnol. 2018;52(1–2):19–26.

[15] Coskun M, Temuz MM. Grafting Studies onto cellulose by atom-transfer radical polymerization. Polym Int. 2005;54:342–7. 10.1002/pi.1684.

[16] Liyana-Pathirana C, Shahidi F. Optimization of extraction of phenolic compounds from wheat using response surface methodology. Food Chem. 2005;93:47–56.

[17] Kumammoto T, Toyooka K, Nishida M, Kuwahara H, Yashimura Y, Kawada J, Kubota S. Effect of 2,4-Dihydro-3H-1,2,4-triazole-3-thiones and thiosemicarbazones on iodide uptake by the mouse thyroid: the relationship between their structure and anti-thyroid activity. Chem Pharm Bull. 1990;38(9):2595–6. 10.1248/cpb.38.2595 1704818

[18] Soares JR, Dins TCP, Cunha AP, Ameida LM. Antioxidant activity of some extracts of Thymus zygis. Free Radical Res. 1997;26:469–78. 9179593 10.3109/10715769709084484

[19] Keser S, Turkoglu S, Celik S, Turkoglu I. Determination of antioxidant capasities of phlomis pungens willd var hispida hub-mor. Asian J Chem. 2012;24:2780–4.

[20] Offeing BM, Martelli S. Steochemistry and antitumour activity of platinium metal complexes of 2-acetypyridine thiosemicarbazones. Transition Met Chem. 1997;22:263–9.

[21] Ferrari BM, Capacchi S, Pelosi G, Reffo G, Tarasconi P, Albertini R, Pinelli S, Lungni P. Synthesis, structural characterization and biological activity of helicin thiosemicarbazone monohydrate and a copper(II) complex of salicylaldehyde thiosemicarbazone. Inorg Chim Acta. 1999;286(2):134–41.

[22] Demir S, Pekdemir S, Keser S, Karatepe A, Koparır M, Karatepe M. Antioxidant and antiproliferative properties of some 2-(4h-[1,2,4] Triazol-3-Yl-sulfanyl-acetamide derivatives. Int J Pure Appl Sci Technol. 2021;7(3):472–9.

[23] Turan N, Topçu MF, Ergin Z, Sandal S, Tuzcu M, Akpolat N, Yilmaz B, Sekerci M. Pro-oxidant and antiproliferative effects of the 1,3,4-thiadiazole–based Schiff base and its metal complexes. Drug Chem Toxicol. 2011;34(4):369–78. 10.3109/01480545.2011.564177. 21714772 10.3109/01480545.2011.564177

[24] Ifuku S, Nomura R, Morimoto M, Saimoto H. Preparation of chitin nanofibers from mushrooms. Materials. 2011;4:1417–25. 28824151 10.3390/ma4081417 PMC5448680

[25] Bojanic V, Jovanovic S, Tabakovic R, Tabakovic I. Synthesis and electrochemistry of grafted copolymers of cellulose with 4-vinylpyridine, 1-vinylimidazole, 1-vinyl-2-pyrrolidinone and 9-vinylcarbazole. J Appl Polym Sci. 1996;60:1719–25.

[26] Haroun AA, Taie HAA. Cytotoxicity and antioxidant activity of beta vulgaris extract released from grafted carbon nanotubes based nanocomposites. Macromol Symp. 2014;337(1):25–33.

[27] Haroun AA, Ahmed EF, El-Halawany NR, Taie HAA. Antimicrobial and antioxidant properties of novel synthesized nanocomposites based on polystyrene packaging material waste. IRBM. 2013;34(3):206–13. 10.1016/j.irbm.2012.12.009.

[28] Haroun AA, Ahmed EF, Abd El-Ghaffar MA. Preparation and antimicrobial activity of poly (vinyl chloride)/gelatin/montmorillonite biocomposite films. J Mater Sci Mater Med. 2011;22(11):2545–53. 21909641 10.1007/s10856-011-4437-x

[29] Kaur H, Rahi DK. Response surface methodology-based optimisation of chitin production and its antioxidant activity from Aspergillus niger. Heliyon. 2024;10(4): e25646. 38404787 10.1016/j.heliyon.2024.e25646 PMC10884427

[30] Limam Z, Selmi S, Sadok S, Abed A. Extraction and characterization of chitin and chitosan from crustacean by-products: Biological and physicochemical properties. Afr J Biotechnol. 2011;10(4):640–7.

[31] Kong C-S, Kim J-A, Ahn B, Byun H-G, Kim S-K. Carboxymethylations of chitosan and chitin inhibit MMP expression and ROS scavenging in human fibrosarcoma cells. Process Biochem. 2010;5(2):179–86.

[32] Yanat M, Colijn I, Schroën K. Chitin nanocrystals provide antioxidant activity to polylactic acid films. Polymers. 2022;14(14):2965. 10.3390/polym14142965. 35890741 10.3390/polym14142965 PMC9320242

[33] Liang TW, Chen YJ, Yen YH, Wang SL. The antitumor activity of the hydrolysates of chitinous materials hydrolyzed by crude enzyme from Bacillus amyloliquefaciens V656. Process Biochem. 2007;42:527–34.

[34] Murata J, Saiki I, Matsuno K, Tokura S, Azumo I. Inhibition of tumor cell arrest in lungs by anti metastatic chitin heparimoid. Jpn J Cancer Res. 1990;80:866–72. 10.1111/j.1349-7006.1990.tb02599.x PMC5918061 2116400

[35] Song Y, Onishi H, Nagai T. Pharmacokinetic characteristics and antitumor activity of the N-succinyl-chitosan-mitomycin C conjugate and the carboxymethyl-chitin-mitomycin C conjugate. Biol Pharm Bull. 1993;16(1):48–54. 10.1248/bpb.16.48. 8369752 10.1248/bpb.16.48

[36] Nishimura K, Nishimura S, Nishi N, Saiki I, Tokura S, Azuma I. Immunological activity of chitin and its derivatives. Vaccine. 1984;2:93–9. 6397928 10.1016/s0264-410x(98)90039-1

[37] Ohya Y, Nonomura K, Ouchi T. In Vivo and in vitro antitumor activity of cm-chitin immobilized doxorubicins by lysosomal digestible tetrapeptide spacer groups. J Bioact Compat Polym. 1995;10(3):223–34. 10.1177/088391159501000302.

